# The challenges in the identification of *Escherichia coli* from environmental samples and their genetic characterization

**DOI:** 10.1007/s11356-022-22870-8

**Published:** 2022-09-12

**Authors:** Adriana Osińska, Ewa Korzeniewska, Agnieszka Korzeniowska-Kowal, Anna Wzorek, Monika Harnisz, Piotr Jachimowicz, Martyna Buta-Hubeny, Wiktor Zieliński

**Affiliations:** 1 Department of Water Protection Engineering and Environmental Microbiology, Faculty of Geoengineering, University of Warmia and Mazury in Olsztyn, Prawocheńskiego 1, 10-720 Olsztyn, Poland; 2 Department of Immunology of Infectious Diseases, Hirszfeld Institute of Immunology and Experimental Therapy, Polish Academy of Sciences, Weigla 12, 53-114 Wroclaw, Poland

**Keywords:** *Escherichia coli*, Virulence genes, MALDI Biotyper, 16S *r*RNA gene sequencing, Gene marker, Wastewater, River water, Antibiotic resistance

## Abstract

*Escherichia coli* bacteria are an essential indicator in evaluations of environmental pollution, which is why they must be correctly identified. This study aimed to determine the applicability of various methods for identifying *E. coli* strains in environmental samples. Bacterial strains preliminary selected on mFc and Chromocult media as *E. coli* were identified using MALDI Biotyper techniques, based on the presence of genes characteristic of *E. coli* (*uid*A, *usp*A, *yai*O), as well as by 16S *r*RNA gene sequencing. The virulence and antibiotic resistance genes pattern of bacterial strains were also analyzed to investigate the prevalence of factors that may indicate adaptation to unsupportive environmental conditions and could have any significance in further identification of *E. coli*. Of the strains that had been initially identified as *E. coli* with culture-based methods, 36–81% were classified as *E. coli* with the use of selected techniques. The value of Cohen’s kappa revealed the highest degree of agreement between the results of 16S *r*RNA gene sequencing, the results obtained in the MALDI Biotyper system, and the results of the analysis based on the presence of the *yai*O gene. The results of this study could help in the selection of more accurate and reliable methods which can be used in a preliminary screening and more precise identification of *E. coli* isolated from environmental samples.

**Supplementary Information:**

The online version contains supplementary material available at 10.1007/s11356-022-22870-8.

## Introduction

*Escherichia coli* are common colonizers of the gastrointestinal tract in humans and warm-blooded animals. These bacteria are gut commensals, but they can also cause intestinal and extraintestinal infections, including urinary tract infections, septicemia, and meningitis in humans and colibacillosis in poultry (Müller et al. 2016). Pathogenic *E. coli* (pathotypes) pose a serious health threat, and *E. coli* infections have high incidence and mortality rates around the world (Poirel et al. 2018). *E. coli* can survive in various environments, including wastewater, soil, water, plants, fruit and vegetables, undercooked meat, and unpasteurized milk (Jang et al. 2017; Devane et al. 2020; Enany et al. 2019). The wide natural host range of *E. coli* can increase the risk of infections caused by this pathogen (Heredia and García 2018). The commensalism or pathogenicity/virulence of *E. coli* can be attributed to the complex balance between the status of the host and the presence and expression of virulence determinants (Raimondi et al. 2019). Strains that cause infections often possess additional traits that facilitate colonization and enable them to avoid the host’s immune system. These traits include adhesion, biofilm formation, toxin production, and avoidance or subversion of host defense mechanisms (Jang et al. 2017). The behavior of *E. coli* has been studied extensively under laboratory conditions, but relatively little is known about this bacterium’s mode of action in the environment (Osińska et al. 2017b).

*E. coli* and its pathotypes are transmitted to the environment with manure, other animal wastes, wastewater, and sewage sludge evacuated from wastewater treatment plants (Osińska et al. 2017a). The presence of *E. coli* in surface water bodies and food products is regarded as an important indicator of recent fecal contamination (Devane et al. 2020). According to some studies, *E. coli* can survive in the environment for long periods of time, and it can integrate with indigenous microbial communities in the environment (Jang et al. 2017). Similarly to the bacteria colonizing the gastrointestinal tract, environmental *E. coli* are influenced mainly by environmental conditions. The survival and growth of environmental *E. coli* are determined by both abiotic (temperature, water and nutrient availability, pH, solar radiation) and biotic factors (ability to acquire nutrients, competition with other microorganisms, biofilm formation) (van Elsas et al. 2011; Jang et al. 2017). Strains characterized by higher genotypic and phenotypic plasticity cope better or adapt more quickly to unsupportive environments (Mallon et al. 2015). The expression of genes encoding resistance to stress was found to be higher in environmental *E. coli* (Vital et al. 2015). *E. coli* has access to the large pool of ARGs in wastewater which is evacuated to surface water bodies with treated effluents. Therefore, *E. coli* present in wastewater can acquire genes from other bacteria in this environment, and they can also significantly enrich the genotype of environmental bacteria through horizontal gene transfer.

In most laboratories that monitor environmental samples, microorganisms are identified with the use of culture-based methods as well as based on their phenotypic traits. However, these methods are not highly reliable because phenotypic characteristics can be unstable, and their expression can be affected by changes in environmental conditions. In addition, biochemical properties do not always entirely reflect the genomic complexity of a given microbial species (Rodrigues et al. 2017). Culture-based methods in microbial diagnostics are also laborious, time-consuming (substrate preparation, dilution, inoculation, incubation, passaging, counting, isolation, and characterization), and burdened with a high risk of incorrect identification. Despite the above, these methods are widely used in the preliminary screening of selected microbial groups due to their ease of application, as well as wide availability and low cost of specific culture media (Franco-Duarte et al. 2019). Microbial analyses are also conducted using MALDI-TOF mass spectrometry, where bacteria, yeast-like fungi, and filamentous fungi are identified based on the presence of ribosomal proteins in cells. This method is applied to pure colonies (a single colony or a liquid culture sample), and the results are generated within minutes. However, MALDI-TOF requires dedicated equipment and software, which substantially increases analytical costs (Siller-Ruiz et al. 2017; Sauget et al. 2017).

Molecular techniques are yet another large group of diagnostic methods that promote rapid and more accurate detection and identification of microorganisms. These techniques are widely used, and they are characterized by high-throughput, high sensitivity, and a short time of analysis. Most molecular methods for bacterial identification rely on DNA analysis, amplification, and sequencing. They include simple techniques that amplify DNA fragments characteristic of a given bacterial species (such as standard PCR, real-time PCR (qPCR), and RAPD-PCR), as well as complex methods based on restriction mapping and directed sequencing of individual genes or the entire genome (Franco-Duarte et al. 2019). Molecular methods for bacterial identification include the detection of species-specific genes, such as genes characteristic for *E. coli* like the *uid*A gene encoding β-D-glucuronidase (Brons et al. 2020), the *yiaO* gene encoding outer membrane protein (Heijnen and Medem 2006), or the *uspA* gene encoding universal stress protein (Molina et al. 2015). Standard PCR can be applied to detect species-specific genes in the DNA of individual pure bacterial colonies or in genomic DNA isolated from environmental samples, which supports the identification of genes in non-culturable or dead cells. Standard PCR is easy to perform, relatively inexpensive, fast, and highly reliable. However, this method does not support the unambiguous identification of microorganisms because it can also produce false-positive results. For example, the *uid*A gene was also detected in several coliform bacteria, including *Citrobacter freundii*, *Enterobacter cloacae*, and *Klebsiella pneumoniae* (Molina et al. 2015). 16S *r*RNA gene sequencing is one of the most sensitive diagnostic methods that is widely used in laboratories. The 16S *r*RNA gene is highly specific for each bacterial species, making it the ideal target in bacterial identification. In this method, the 16S *r*RNA gene is amplified and sequenced, and the obtained nucleotide sequences are identified by comparison with those deposited in databases (Clifford et al. 2012). 16S *r*RNA gene sequencing is not only highly sensitive but also repeatable and accurate; therefore, it is regarded as the gold standard for microbial identification at the species level. However, sequencing costs are still relatively high. In addition, sequencing is often outsourced to specialist laboratories, which prolongs wait times for the results (Buszewski et al. 2017).

This study aimed to evaluate various methods for identifying *E. coli* strains isolated from the environment (wastewater and river water). Bacteria were identified with the use of culture-based methods, molecular techniques for detecting species-specific genes characteristic for *E. coli* (*uid*A, *yai*O, *usp*A), MALDI-TOF, and 16S *r*RNA gene sequencing. Clonal relatedness between isolates was determined by ERIC PCR, and the phylogenetic lineage of selected *E. coli* isolates was inferred with the use of the grouping method described by Clermont et al. (2000). Moreover, to investigate the prevalence of factors that may indicate adaptation to unsupportive environmental conditions and may be connected with increased pathogenicity, isolates were tested for various antibiotic resistance (number of tested genes 13) and virulence determinant (number of tested genes 9) genes pattern.

## Materials and methods

### Sampling sites and sample collection

Samples of untreated wastewater (UWW) and treated wastewater (TWW) and samples of river water collected upstream (URW) and downstream (DRW) from the wastewater discharge point were collected in four small municipal wastewater plants (which do not process hospital wastewater) in the Region of Warmia and Mazury in Poland (Osińska et al. 2019, 2020). Samples were collected in two periods of the year: in October 2018 (autumn season) and February 2019 (winter season). A total of 16 river water samples and 16 wastewater samples were collected for analysis. Samples of wastewater and river water were collected into sterile bottles, transported to the laboratory at a temperature of 4 °C, and processed on the day of collection.

### Selection of presumptive Escherichia coli strains

The collected samples of untreated wastewater were diluted in 0.85% NaCl to obtain individual colonies. Samples, in which bacterial counts were expected to be low (treated wastewater, river water), were passed through a cellulose filter (47 mm in diameter; 0.45-μm pore size; Millipore). In a preliminary analysis, all samples were incubated in parallel on mFc agar and Chromocult coliform agar (both media from Merck, Germany) in Petri dishes at a temperature of 44.5 ± 0.2 °C for 24 h and 36.0 ± 2 °C for 21 ± 3 h, respectively (Grabow et al. 1981; Wohlsen 2011).

Individual characteristic dark blue colonies (a total of 384 strains: from river water, 180 strains, and from wastewater samples, 204 strains) from mFc agar and dark-blue to violet colonies growing on Chromocult coliform agar (a total of 365 strains, including 178 strains from river water samples and 187 strains from wastewater samples) were identified as potential *E. coli*. At least ten characteristic colonies from each kind of sample and media (mFc agar and Chromocult coliform agar) have been chosen for further analysis. However, due to the low number of presumptive *E. coli* isolates in the river water samples, it was not possible for all samples. The isolates from mFc agar were transferred to plates containing Chromocult coliform agar and isolates from Chromocult coliform agar were transferred to plates containing mFc agar for additional identification to each other. A total of 305 strains (including 148 strains from river water samples and 157 strains from wastewater samples) confirmed on both media as presumptive *E. coli* were selected for further analysis. Colonies that were preliminarily identified as *E. coli* on both selective media were passaged on LB agar (Merck, Germany) plates and used in successive analyses. The isolates were stored in Miller’s LB broth (Merck, Germany) supplemented with glycerol (10%) at a temperature of – 80 °C.

### Genomic DNA extraction

To extract genomic DNA, a loopful of bacterial colonies harvested from agar plates was suspended in 0.5 mL of sterile water, heated at 95 °C for 10 min, and centrifuged at 5000 rpm for 5 min at 4 °C. The concentration and quality of the extracted DNA were determined with a Multiskan™ Sky (Thermo Fisher Scientific Inc., USA) spectrophotometer. Genomic DNA was stored at −20 °C for further analysis. The quality of the analytical process was controlled with ATCC standard strains of *E. coli* (ATCC25922). To control the presence of antibiotic resistance and virulence genes in tested microorganisms, *E. coli* strains where the analyzed genes had been previously confirmed by the authors (Korzeniewska et al. 2013; Osińska et al. 2017a) were used.

Lines 166-168: what do you mean by “the presence of antibiotic resistance and virulence genes was determined in E. coli strains where the analyzed genes had been previously confirmed by the authors”? Does it mean that the presence of genes in these *E. coli* isolates were already confirmed in the previous studies, or *E. coli* isolates were tested using the methods developed in the previous studies?

### Identification of Escherichia coli: MALDI-TOF identification

A total of 305 strains, including 148 strains from river water samples and 157 strains from wastewater samples, were analyzed. Bacterial samples were prepared according to the manufacturer’s protocol (Bruker Daltonics, Bremen, Germany). Two to five bacterial colonies of each strain were suspended in water and precipitated with ethanol. After drying, equal volumes of 70% formic acid and acetonitrile were added. After centrifugation (13,000 × g, 2 min), 1 μl of the supernatant was transferred to a ground steel MALDI plate for analysis, with α-cyano-4-hydroxy-cynnamic acid in 50% acetonitrile and 2.5% TFA as the matrix. Bacterial strains were identified with the use of the ultrafleXtreme MALDI-TOF mass spectrometer and the MALDI Biotyper classification software (Bruker Daltonics, Bremen, Germany). Spectra were recorded in positive linear mode for a mass range of 2000–20 000 Da. Each spectrum was obtained by averaging 1500 laser shots acquired in automatic mode in flexControl v. 3.4 software (Bruker Daltonics, Bremen, Germany). The spectra were externally calibrated using an *E. coli* DH5-alpha standard (Bruker Daltonics, Bremen, Germany). Bacterial isolates were identified in MALDI Biotyper v. 3.1 (MSP 6904) classification software (Bruker Daltonics, Bremen, Germany).

### Identification based on taxonomic genes

The DNA isolated from potential *E. coli* strains (305 strains) was analyzed for the presence of *uid*A, *yai*O, and *usp*A genes by standard PCR using dedicated primers to confirm the taxonomic identity of these microorganisms. Strains harboring species-specific genes were classified as *E. coli*, whereas strains, where species-specific genes were absent, were classified as non–*E. coli.* All primers had been previously validated (refer to “Supplementary information,” Table S1, for primer sequences, amplicon sizes, annealing temperatures, references for each sequence, and additional details regarding PCR conditions). The products were separated by electrophoresis on 2% agarose gel (Sigma-Aldrich, Merck, Germany) stained with ethidium bromide (0.5 μg/mL) and were visualized in Gel Doc EZ (Bio-Rad, USA).

### Identification based on sequencing results

All the isolates were identified by 16S *r*RNA gene sequencing. Universal primers 27F and 1492R were used to amplify nearly full-length 16S *r*RNA gene sequences according to a previously described method (Gillan et al. 1998). After amplification, DNA was separated by electrophoresis on an agarose gel stained with ethidium bromide (0.5 μg/mL). The exact 16S *r*RNA sequence was determined when the PCR product was proper. The PCR amplicons were purified and sequenced by applying both forward and reverse primers in amplification (Genomed S.A., Poland). The obtained sequences were identified using the BLAST program available on the website of the National Center for Biotechnology Information.

### Clonal analysis by ERIC PCR

ERIC-PCR fingerprinting was performed to determine the clonal relatedness of selected *E. coli* isolates. The analysis involved 238 strains (110 strains from river water samples and 128 strains from wastewater samples) which were identified as *E. coli* based on the results of 16S *r*RNA gene sequencing. The ERIC-PCR approach relies on primers complementary to 124–127 bp repetitive sequences in the bacterial genome which contain highly conserved sequences of approximately 44 bp in the center (Asgarani et al. 2015). ERIC PCR was conducted according to Versalovic et al. (Versalovic et al. 1991) with primers ERIC 1 and ERIC 2 (Supplementary information, Table S1). Gel electrophoresis was performed as mentioned above. Optimization and band position tolerance were set at 1%. The similarity between fingerprints was calculated with the Dice coefficient. Cluster analysis was performed using the unweighted pair-group method (UPGMA) with average linkages. The similarities in the profiles of the identical isolates that were analyzed in separate experiments and compared in different gels ranged from 98 to 100%. Some *E. coli* isolates had similar profiles in ERIC PCR fingerprinting. However, similar isolates which originated from different samples (different sampling sites and seasons) were included in further microbial analysis.

### Identification of virulence determinant genes characteristic of Escherichia coli

The following virulence markers were analyzed: *eae* (attaching and effacing lesions, intimin encoding gene), *bfp*A (localized adherence, encoding the production of type IV pili), CVD432 gene encoding proteins responsible for enteroaggregative adherence, *ipa*H (enteroinvasive mechanism, responsible for adhesion to and colonization of epithelial cells), LT gene encoding the heat-labile toxin (activates adenylyl cyclase on the surface of epithelial cells and disrupts ion pump function), the heat-stable toxin (ST) gene (activates guanylyl or adenylyl cyclase on the cell surface and induces ion outflow from cells), *stx*1 and *stx*2 (Shiga toxins, inhibit protein synthesis and induce cell apoptosis), *iro*N (catecholate siderophore receptor gene), *fim*H (type 1 fimbriae), *sfa* (fimbrial adhesin), *hly*D (transport gene of the hemolysin operon) and *pap*C (pilus assembly).

The presence of 13 virulence determinant genes characteristic of *E. coli* (*bfp*A, *eae*, CVD43, LT, ST, *stx*1, *stx*2, *ipa*H, *iro*N, *fim*H, *sfa*, *hly*D, *pap*C) was determined in the DNA isolated from bacterial strains during standard PCR. The DNA of strains identified as *E. coli* in 16S *r*RNA gene sequencing, i.e., 110 strains from river water samples and 128 from wastewater samples, was used in the analysis. The genomic DNA of strains identified as non–*E. coli,* i.e., 38 strains from river water samples and 29 strains from wastewater samples, was also analyzed for the presence of virulence genes characteristic of *E. coli.* Primer sequences and the expected size of PCR products are presented in the “Supplementary information” (Table S1). Gel electrophoresis was performed as mentioned above

### Phylogenetic analysis

The membership to particular phylogroup of the identified *E. coli* strains was inferred with the use of the multilocus sequence typing (MLST) technique based on the method described by Clermont et al. (Clermont et al. 2000), where two DNA markers (*chu*A and *yja*A) and *tsp*E4.C2 DNA sequences are used to classify *E. coli* isolates into one of the four phylogroups of a phylogenetic tree.

### Antimicrobial susceptibility of Escherichia coli and non–E. coli

The sensitivity of bacterial isolates to β-lactam and tetracycline antibiotics which are the most used in human and animal treatment in the world (ECDC 2020) was tested using culture-based and molecular methods. Isolates resistant to β-lactams (ampicillin, cefuroxime) and tetracyclines (oxytetracycline, doxycycline) were determined in plates containing TSA medium (Oxoid, UK) with the addition of (a) ampicillin (8 μg/mL), (b) cefuroxime (8 μg/mL), (c) oxytetracycline (16 μg/mL), and (d) doxycycline (16 μg/mL). Both *E. coli* (*n*=238) and non–*E. coli* (*n*=67) strains were analyzed. The antimicrobial dose was determined according to EUCAST (EUCAST European Committee on antimicrobial susceptibility testing 2014) and CLSI (CLSI 2015) guidelines. Microorganisms were incubated at 37 °C for 48 h. The presence of five tetracycline resistance genes (*tet*A, *tet*B, *tet*M, *tet*K, *tet*L) and four β-lactam resistance genes (*bla*_TEM,_ *bla*_SHV_, *bla*_OXA,_ *bla*_CTX-M_) in the DNA of bacterial isolates were determined by standard PCR (Supplementary information, Table S1). Gel electrophoresis was performed as mentioned above.

### Statistical analysis

Statistical analyses were carried out in R Studio (v. 1.2.1335, R Development Core Team, New Zealand) at a significance level of *p* < 0.05. Cohen’s kappa was calculated to measure the degree of agreement between the results of sequencing analyses and the other methods for identifying *E. coli*. In analyses that rely on genetic markers, bacterial strains were identified only as *E. coli* or non–*E. coli*; therefore, the results of 16S *r*RNA gene sequencing and the results generated by the MALDI Biotyper system were also classified as *E. coli* or non–*E. coli.* A neighbor-joining dendrogram (based on the 16S *r*RNA nucleotide sequence of *E. coli* and isolate characteristics) was developed based on the origin, antibiotic resistance profile and the presence of antibiotic resistance determinants to determine the relatedness of selected isolates. A phylogenetic tree of the analyzed isolates was built in Molecular Evolutionary Genetics Analysis software (MEGA7) (Kumar et al. 2016).

## Results and discussion

### Identification of Escherichia coli

**Fig. 1:**
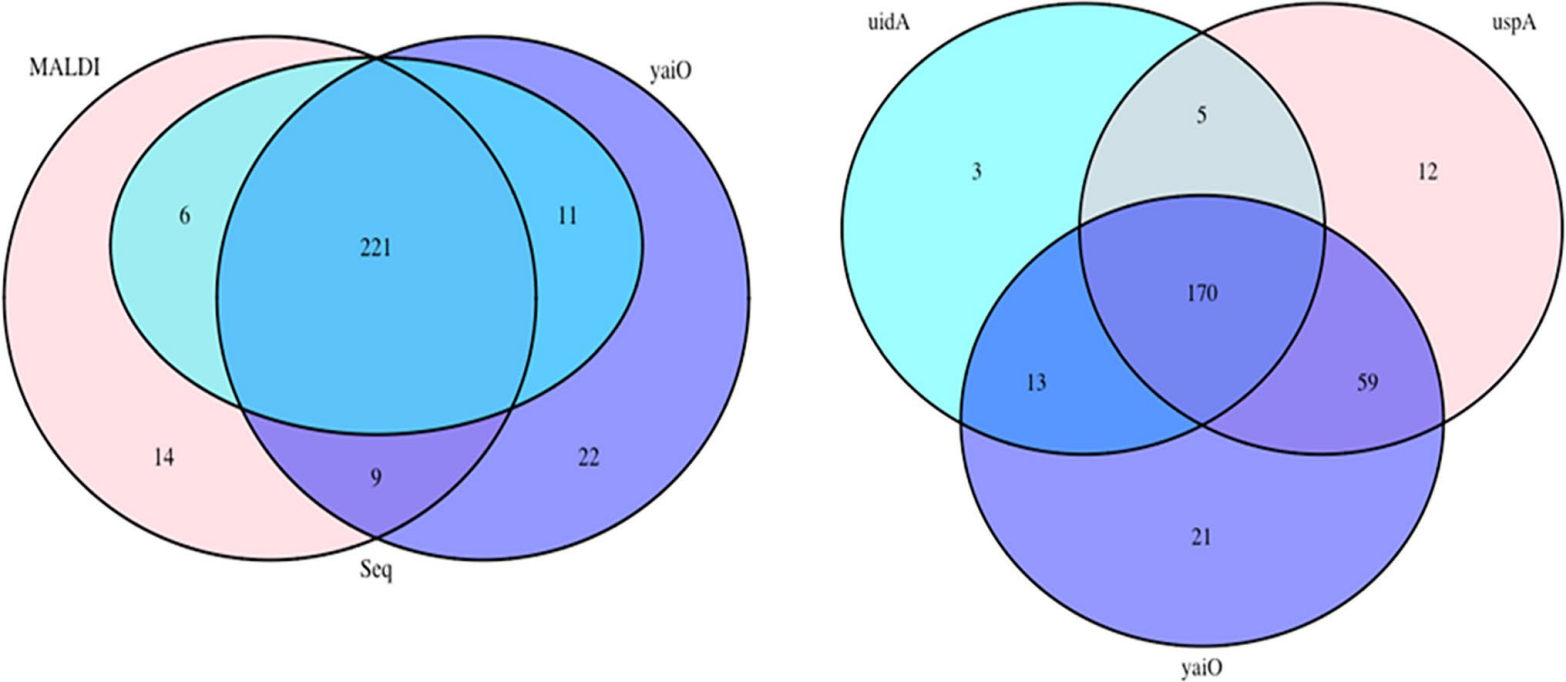
Venn diagram illustrating the number of strains identified as *E. coli* with the applied microbial detection techniques

From the group of randomly selected strains from both mFc agar and Chromocult medium that displayed features characteristic of *E. coli*, a total of 305 strains, including 148 strains from river water samples and 157 strains from wastewater samples, were selected for further analysis. The following number of strains were identified as *E. coli* with the use of the applied microbial identification techniques: MALDI Biotyper, 250 strains; 16S *r*RNA gene sequencing, 238 strains; presence of the *uid*A gene, 110 strains; presence of the *usp*A gene, 122 strains; and presence of the *yai*O gene, 132 strains (Fig. 1). The Venn diagram (Fig. 1) has been used to illustrate the number of common identical results obtained using different methods.

Depending on the identification method, the identity of 36–81% of the strains initially identified as *E. coli* using the culture-based method was confirmed. Among the most popular microbial identification techniques, i.e., biochemical tests, MALDI-TOF, standard PCR, DNA microarray and whole-genome sequencing, bacterial identification using selective media is least expensive and least complex to perform (Váradi et al. 2017). However, selective culture media are characterized by low specificity and sensitivity compared to molecular biology technique. Therefore, selective culture media are not a highly reliable method for identifying bacteria from diverse environments, and the results have to be confirmed with more accurate techniques. However, due to low cost and simplicity of use, selective culture media appear to be a robust screening method for the preliminary selection of bacteria required for further molecular analyses.

Since 16S *r*RNA gene sequencing is generally regarded as the most accurate and reliable identification method, the accuracy of the remaining diagnostic techniques, i.e., MALDI Biotyper and analyses of molecular markers (*uid*A, *usp*A, *yai*O), was verified by comparing the results obtained by the above methods with the results of 16S *r*RNA gene sequencing. The values of Cohen’s kappa revealed that the results produced by the MALDI Biotyper system and the PCR analysis of the *yai*O gene were most consistent with the outcomes of 16S *r*RNA gene sequencing (Table 1).

**Table 1:** Degree of agreement between the results of 16S *r*RNA gene sequencing (Seq) and the remaining molecular identification methods based on Cohen’s kappa

	Strains from river water	Strains from wastewater
	*Kappa*	*Kappa*
Seq vs MALDI	0.65	0.65
Seq vs *uid*A	0.44	0.499
Seq vs *usp*A	0.5	0.479
Seq vs *yai*O	0.6	0.57

Landis and Koch (Landis and Koch 1977) identified the following groups based on the values of Cohen’s kappa: no agreement (kappa <0), slight (0–0.20), fair (0.21–0.40), moderate (0.41–0.60), substantial (0.61–0.80), and perfect (0.81–1.0). Based on these assumptions and the calculated values of Cohen’s kappa, it was concluded that none of the identification methods applied in this study was highly consistent with the results of 16S *r*RNA gene sequencing because “perfect” agreement was not achieved in any of the cases. The strains from river water samples identified by MALDI Biotyper and based on the presence of the *yai*O gene were characterized by “substantial” agreement with the results of 16S *r*RNA gene sequencing. For the strains from wastewater samples, only MALDI Biotyper results exhibited “substantial” agreement with the results of 16S *r*RNA gene sequencing. In contrast, the degree of agreement in the remaining methods was “moderate.” However, Cohen’s kappa was significantly higher in the analysis based on the presence of the *yai*O gene than in the analyses based on the remaining genetic markers (*uid*A and *usp*A). In a study of bacterial communities from copper mining samples, Avanzi et al. (Avanzi et al. 2017) also reported the high agreement (82% of identified bacteria) between the results of MALDI Biotyper and 16S rDNA sequencing. They also noted that the limitations of the MALDI Biotyper technique can be attributed to the high genetic diversity of environmental bacteria which were compared against a relatively small database in the MALDI Biotyper classification software. Despite these limitations, high rates of identification were achieved in the MALDI Biotyper system.

**Fig. 2:**
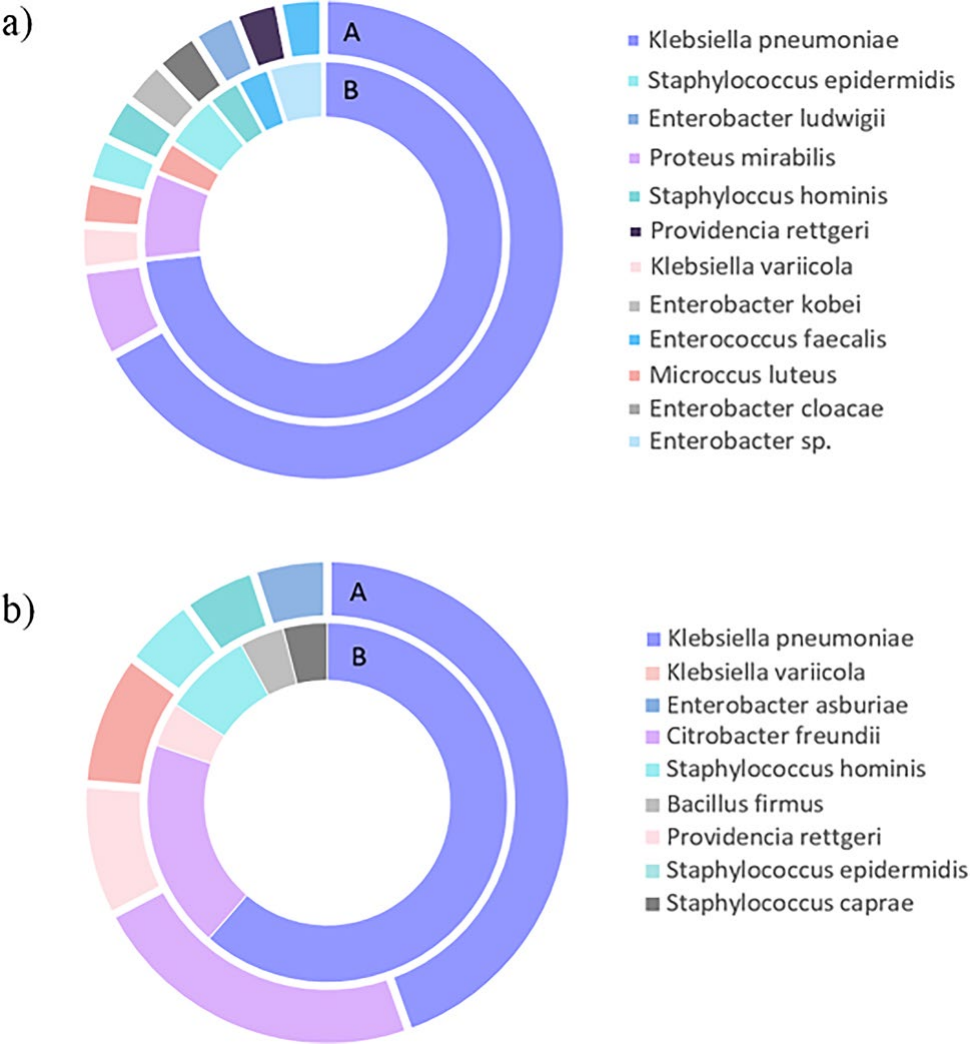
Percentage of bacteria identified as non–*E. coli* in samples of **a** river water and **b** wastewater. **A** MALDI Biotyper; **B** 16S *r*RNA gene sequencing

Unlike methods based on the presence of specific genetic markers, 16S *r*RNA gene sequencing and MALDI Biotyper support more accurate microbial identification and classification at the genus and/or species level. In the group of strains isolated from river water samples, 22% and 26% of all strains selected for the study (*n*=305) were classified as non–*E. coli* based on the MALDI Biotyper analysis and 16S *r*RNA gene sequencing, respectively. *Klebsiella pneumoniae* and *Proteus mirabilis* were the predominant non–*E. coli* bacteria in river water samples (Figure 2a). In the MALDI Biotyper analysis, 67% and 6% of all *non–E. coli* strains were classified as *K. pneumoniae* and *P. mirabilis,* respectively, whereas in 16S *r*RNA gene sequencing, these species accounted for 74% and 8% of all *non–E. coli* strains, respectively.

The percentage of strains identified as *non–E. coli* was much lower in wastewater samples than in river water samples (Fig. 1), and it was determined at 14% and 16% of all strains selected for the study based on the MALDI Biotyper analysis and 16S *r*RNA gene sequencing, respectively. *K. pneumoniae* was also the predominant species in the group of *non–E. coli* strains isolated from wastewater samples, and it accounted for 45% and 62% of all non–*E. coli* identified by MALDI Biotyper and 16S *r*RNA gene sequencing, respectively (Fig. 2b).

The percentage of *K. pneumoniae* among non–*E. coli* strains were much lower in isolates from wastewater samples than in isolates from river water samples. In wastewater samples, *Citrobacter freundii* accounted for 23% and 19% of all non–*E. coli* strains were identified by the MALDI Biotyper analysis and 16S *r*RNA gene sequencing, respectively.

### Prevalence of virulence genes characteristic for Escherichia coli

**Fig. 3:**
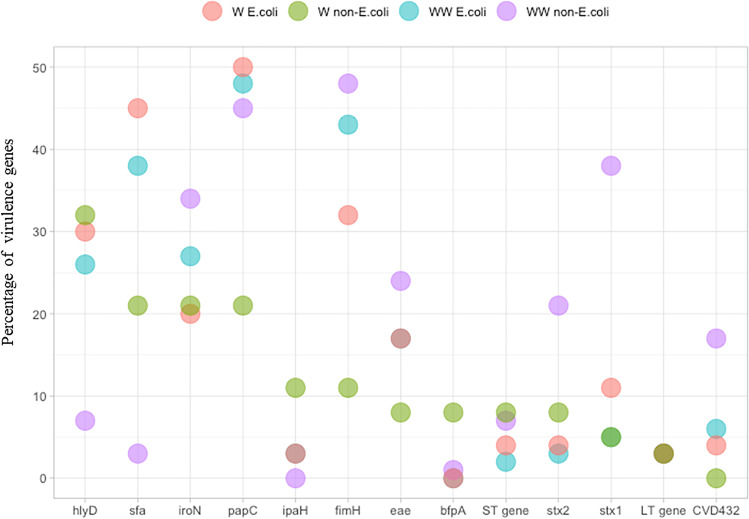
Prevalence of virulence genes in *E. coli* and non–*E. coli* strains. W *E. coli*, *E. coli* strains from river water samples; W non–*E. coli*, non–*E. coli* strains from river water samples; WW *E. coli, E. coli* strains from wastewater samples; WW non–*E. coli,* non–*E. coli* strains from wastewater samples.

The virulence genes were studied to investigate the prevalence of factors that may indicate adaptation to unsupportive environmental conditions and whose could have any significance in further identification of *E. coli*. The *pap*C gene was the most prevalent virulence gene that was detected in 50% of *E. coli* strains from river water samples, and in 48% *E. coli* strains from wastewater samples (Fig. 3, Fig. S1, Fig. S2).

The following genes were also frequently noted in *E. coli* strains from river water and wastewater samples: *fim*H (32% and 43% strains, respectively), *sfa* (45% and 38% strains, respectively), and *hly*D (30% and 26% strains, respectively). The presence of CVD432, LT, ST, *ipa*H, and *stx*2 genes were detected in less than 5% of the strains isolated from river water and wastewater. None of the examined *E. coli* strains harbored the *bfp*A gene. In contrast, Osińska et al. (Osińska et al. 2017b) reported that *bfp*A was the most prevalent virulence gene in *E. coli* strains, which was present in more than 60% of the strains isolated from both wastewater and river water samples. Such a high prevalence of the *bfp*A gene in *E. coli* strains could be attributed to the fact that the analyzed samples were a mixture of municipal and hospital wastewater, where this gene is more frequently noticed. In the present study, *E. coli* strains were isolated from municipal wastewater only. El-Shaer et al. (El-Shaer et al. 2018) reported that *fim*H was the most common virulence gene that was identified in around 90% of environmental isolates. They also found that *stx*2 and *hly*A were more prevalent in environmental than in clinical strains.

No significant differences were found in the prevalence of virulence genes between *E. coli* strains isolated from river water and wastewater samples (Fig. S1, Fig. S2). However, *fim*H was more frequently noted (by 10%) in *E. coli* strains from wastewater samples, whereas *sfa* was more prevalent (by 6%) in river water samples. Wastewater and river water samples also differed in the prevalence of *stx*1 and *iro*N genes (by 6%). The *stx*1 gene was more frequently identified in *E. coli* strains isolated from wastewater samples, whereas *iro*N was more prevalent in *E. coli* strains isolated from river water samples. Bacterial strains harboring virulence genes, in particular genes characteristic of uropathogenic *E. coli* (UPEC), such as *fim*H and *pap*C, are observed mainly in the hospital environment (El-Shaer et al. 2018). However, the presence of virulence genes was observed in *E. coli* strains isolated from both wastewater samples (Zhang et al. 2016; Jiang et al. 2019) and other environmental samples (Osińska et al. 2018; Pérez-Etayo et al. 2020), including in samples of drinking water (Moglad et al. 2020). The absence of differences in the prevalence of virulence genes between strains isolated from wastewater and water samples could be indicative of gene transmission between bacteria colonizing different environments. According to Anastasi et al. (Anastasi et al. 2012), strains harboring virulence genes are more likely to survive the wastewater treatment process, including disinfection. These strains are evacuated to surface water bodies with treated effluence, and they can disseminate virulence genes to environmental bacteria via horizontal gene transfer (Bengtsson-Palme et al. 2018).

Virulence genes characteristic of *E. coli* were also detected in non–*E. coli* strains. Carneiro et al. (Carneiro et al. 2017) observed the presence of *fim*H, *pap*C, and *hly*D virulence genes, which are usually detected in *E. coli,* also in *K. pneumoniae* strains from fecal samples. However, the prevalence of most virulence genes differed between non–*E. coli* and *E. coli* strains. Six of the 13 analyzed virulence genes were more frequently noted in non–*E. coli* than in *E. coli* strains from river water samples, and the prevalence of nine virulence genes was higher in non–*E. coli* than in *E. coli* strains from wastewater samples. The *bfp*A gene was not identified in any *E. coli* strains, but it was detected in 8% of non–*E. coli* strains isolated from river water samples and in 10% of non–*E. coli* strains isolated from wastewater samples. In the group of non–*E. coli* strains from wastewater samples, the prevalence of Shiga toxin genes *stx*1 (38% of non–*E. coli* strains) and *stx*2 (21% of non–*E. coli* strains) was also considerably higher than in *E. coli* strains from wastewater samples, where *stx*1 was identified in 5% of the strains and *stx*2 was detected in only 3% of the strains. Shiga toxins are produced mainly by *E. coli* and *Shigella dysenteriae,* but they are also synthesized by other bacteria of the family *Enterobacteriaceae,* including *Citrobacter freundii*, *Enterobacter cloacae*, and *Shigella flexneri* (Herold et al. 2004; Tajeddin et al. 2020). Shiga toxins are encoded by bacteriophages, which is why they are highly mobile and can be easily transferred between bacteria (Bai et al. 2018). The production of type IV bundle-forming pili (BFP) is also a characteristic feature of *E. coli,* but these fimbriae are also produced by other Gram-negative pathogens (Blank et al. 2000).

In the MLST phylogenetic classification analysis based on the protocol designed by Clermont et al. (Clermont et al. 2000), the highest percentage of *E. coli* strains from river water samples were assigned to groups B1 (33% of all *E. coli* strains from river water samples) and D2 (17% of all *E. coli* strains from river water samples) (Table 2). The highest percentage of *E. coli* strains from wastewater samples were assigned to groups A1 (25% of all *E. coli* strains from wastewater samples) and B1 (25% of all *E. coli* strains from wastewater samples). The smallest percentage of *E. coli* strains isolated from both river water and wastewater samples were assigned to group B2. In a study by El-Shaer et al. (El-Shaer et al. 2018), the highest percentage of environmental strains were also assigned to phylogenetic groups B1 (60.6% of isolates), A (24.2%), B2, and D (6.1%). Pérez-Etayo et al. (Pérez-Etayo et al. 2020) reported that the majority of strains assigned to phylogenetic groups B1, B2, and D were clinical and highly virulent isolates. In their study, the majority of strains from water and wastewater samples were classified to group A or group B1, but some strains were also assigned to phylogenetic groups B2 and D.

**Table 2:** Percentage of *E. coli* strains assigned to different phylogenetic groups

	A0	A1	B1	B22	B23	D1	D2	Non-typeable
*E. coli* from river water	8%	16%	33%	2%	12%	12%	17%	0%
(*n*=110)	(9)	(18)	(36)	(2)	(13)	(13)	(19)	(0)
*E. coli* from wastewater	9%	25%	25%	3%	6%	15%	15%	2%
(*n*=128)	(11)	(33)	(32)	(4)	(8)	(19)	(19)	(2)

### Antimicrobial susceptibility of E. coli and non–E. coli

The antimicrobial susceptibility of *E. coli* and non–*E. coli* strains were determined based on phenotype and the prevalence of genes encoding resistance to different antibiotic groups. The obtained result allowed us to differentiate the analyzed isolates (*E. coli* vs non–*E. coli*) and identify drug resistance genes characteristic of a given group of strains.

Most *E. coli* strains were resistant to β-lactams and tetracyclines (Fig. S1, Fig. S2). Antibiotic resistance was more frequently observed in *E. coli* strains isolated from wastewater samples than from river water samples (Table 3). More than 94% and 95% of *E. coli* strains isolated from river water and wastewater samples, respectively, were resistant to ampicillin, whereas 81% and 86% of these strains were resistant to oxytetracycline, respectively. *E. coli* strains resistant to cefuroxime were less prevalent, but they accounted for nearly 50% and 57% of the strains isolated from river water and wastewater, respectively. Old-generation antibiotics such as penicillin and tetracyclines are widely used, and bacterial strains resistant to these antimicrobials are ubiquitous in the environment. In the current study, at least 83% of non–*E. coli* strains isolated from wastewater samples were resistant to three out of the four tested antibiotics, and all strains were resistant to ampicillin. Antibiotic resistance was less frequently noted in non-*E. coli* strains from river water than from wastewater samples. Despite the above, nearly 84% of non–*E. coli* strains from river water were resistant to ampicillin, and 51% were resistant to oxytetracycline. In a study by Osińska et al. (Osińska et al. 2020), the highest percentage of antibiotic-resistant *E. coli* were insensitive to ampicillin, including 88% of the strains from wastewater samples and 82% of the isolates from river water samples. In contrast to the present findings, Osińska et al. (Osińska et al. 2020) did not report an equally high percentage of bacteria resistant to tetracycline and found that *E. coli* strains resistant to tetracycline accounted for up to 22% and 50% of all *E. coli* bacteria isolated from wastewater and river water samples, respectively. Enany et al. (Enany et al. 2019) observed that *E. coli* strains isolated from environmental and avian sources were highly resistant to ampicillin.

**Table 3:** Percentage of antibiotic-resistant *E. coli* and *non–E. coli* strains from river water and wastewater samples. *OX* oxytetracycline; *DOX* doxycycline; *AMP* ampicillin; *CXM* cefuroxime

		OX	DOX	AMP	CXM
River water	*E. coli*	81%	71%	94%	50%
	(*n*=110)	(89)	(78)	(103)	(55)
	Non–*E. coli*	51%	43%	84%	41%
	(*n*=37)	(19)	(16)	(31)	(15)
Wastewater	*E. coli*	86%	80%	95%	57%
	(*n*=128)	(110)	(103)	(121)	(73)
	non–*E. coli*	97%	83%	100%	48%
	(*n*=29)	(28)	(24)	(29)	(14)

**Fig. 4:**
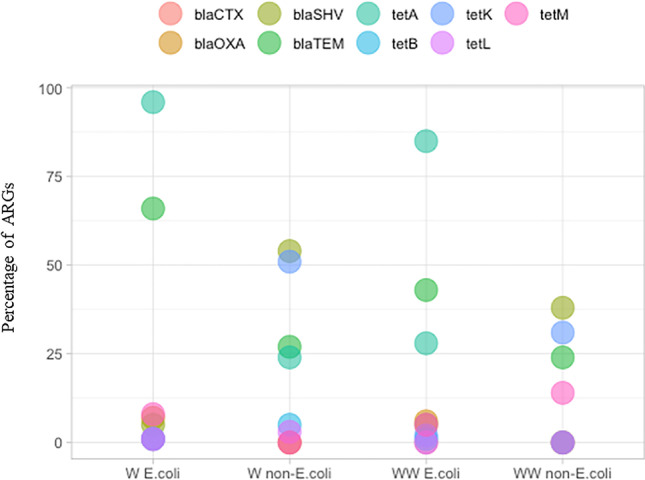
Percentage of *E. coli* and non–*E. coli* strains harboring antibiotic resistance genes. W *E. coli*, *E. coli* strains from river water samples; W non–*E. coli*, non–*E. coli* strains from river water samples; WW *E. coli*, *E. coli* strains from wastewater samples; WW non–*E. coli*, non–*E. coli* strains from wastewater samples.

*E. coli* isolated from both wastewater and river water samples were characterized by a high prevalence of *tet*A and *bla*_TEM_ genes (Fig. 4, Fig. S1, Fig. S2). The *tet*A and *bla*_TEM_ genes were identified in 96% and 66% of *E. coli* strains from river water samples, respectively, and in 85% and 43% of *E. coli* strains from wastewater samples, respectively. The remaining antibiotic resistance genes were detected in less than 8% of *E. coli* strains from both river water and wastewater samples. None of the *E. coli* strains isolated from wastewater samples harbored *tet*B or *bla*_CTX_ genes. Osińska et al. (Osińska et al. 2017b) also observed that *bla*_TEM_ was the most frequent β-lactam resistance gene in *E. coli* strains isolated from water and wastewater samples. In the cited study, *tet*A was the most prevalent tetracycline resistance gene.

The prevalence of the analyzed antibiotic resistance genes was higher in non–*E. coli* than in *E. coli* strains. Non–*E. coli* strains most frequently harbored *bla*_SHV_ and *tet*K genes which were detected in 38% and 31% of the strains from wastewater samples, respectively, and in 54% and 51% of the strains from river water samples, respectively. In a study by Carnerio et al. (Carneiro et al. 2017), the *bla*_SHV_ was also more frequently identified in *K. pneumoniae* than in *E. coli* strains. *tet*A and *bla*_TEM_ were most prevalent in *E. coli* strains, but they were noted in less than 25% of non–*E. coli* strains from both wastewater and water samples. Non–*E. coli* strains isolated from water samples did not harbor *tet*M, *bla*_CTX_ and *bla*_OXA_ genes, whereas *tet*B, *tet*M, *tet*L, and *bla*_CTX_ genes were not identified in non–*E. coli* strains isolated from wastewater samples. The presence of genes encoding the production of β-lactamase enzymes is the main mechanism of antibiotic resistance in Gram-negative pathogens, which is commonly encountered in *E. coli*. TEM and CTX-M enzymes from the group of extended-spectrum β-lactamases (ESBL) are encoded mostly on mobile genetic elements and are readily transmitted among *Enterobacteriaceae* (Cag et al. 2016). Therefore, it cannot be reliably ascertained that the prevalence of the analyzed drug resistance genes plays a significant role in the identification and differentiation of *E. coli* and non-*E. coli* strains. The frequency of antimicrobial resistance genes is influenced by a large number of environmental variables, which is why it cannot be a reliable tool for the identification of environmental *E. coli* strains*.*

## Conclusions

The results of this study confirm that culture-based methods involving selective media do not support the explicit identification of *E. coli* strains isolated from environmental samples. Therefore, these methods can be used only for preliminary screening of microorganisms, and their results have to be validated by at least one analytical technique using species-specific genetic markers. However particularly recommended methods for microbial identification are MALDI-TOF method and/or 16S rRNA gene sequencing, due to their high accuracy and reliability. The most prevalent virulence gene was *pap*C, which encodes P fimbriae. However, the frequency of *pap*C and the remaining virulence genes did not differ significantly between *E. coli* strains isolated from river water and wastewater samples. Additionally, also among the remaining virulence genes presence of virulence genes was not dependent on where the *E. coli* strains were obtained and did not affect strain differentiation in the identification conducted. Moreover, we observed that most *E. coli* strains from river water and wastewater samples harbored genes that encode resistance to ampicillin (*bla*_TEM_) and oxytetracycline (t*etA*). This study has revealed a high prevalence of virulence determinant and antibiotic resistance genes in *E. coli* strains isolated from environmental samples. In particular, the occurrence of virulence genes associated with different *E. coli* pathotypes in strains from river water pose a direct threat to the health and lives of humans and animals using surface water bodies. Finally, this suggests that water bodies which received treated wastewater should be monitored not only for the occurrence of *E. coli* but also screening for virulence and antibiotic resistance genes in these strains.

## Supplementary information

ESM 1 Table S1: PCR primers and conditions used in this study, Fig. S1: Phylogenetic trees of E.coli strains isolated from river water (I-IV – sampling sites – the river which receive effluents from WWTP; 1-28 – number of strains isolated from each sampling site), Fig. S2: Phylogenetic trees of E.coli strains isolated from wastewater (I-IV – sampling sites – the WWTPs; 1-28 – number of strains isolated from each sampling site). (DOCX 939 kb)

## Data Availability

All data generated and analyzed during our study are included in this article.
