# Supplementary material for: The challenges in the identification of Escherichia coli from environmental samples and their genetic characterization
Source: Environ Sci Pollut Res Int. 2022 Sep 12;30(5):11572–83. doi: 10.1007/s11356-022-22870-8 (PMC9898413; doi:10.1007/s11356-022-22870-8)
Supplement: Supplementary file 1 — Table S1: PCR primers and conditions used in this study, Fig. S1: Phylogenetic trees of E.coli strains isolated from river water (I-IV – sampling sites – the river which receive effluents from WWTP; 1-28 – number of strains isolated from each sampling site), Fig. S2: Phylogenetic trees of E.coli strains isolated from wastewater (I-IV – sampling sites – the WWTPs; 1-28 – number of strains isolated from each sampling site). (DOCX 939 kb) [file 11356_2022_22870_MOESM1_ESM.docx]

**The challenges in the identification of *Escherichia coli* from environmental samples and their genetic characterization**

*Adriana Osińska ^a^, Ewa Korzeniewska ^a,^*, Agnieszka Korzeniowska-Kowal ^b^, Anna Wzorek ^b^, Monika Harnisz ^a^, Piotr Jachimowicz ^a^, Martyna Buta-Hubeny ^a^ and Wiktor Zieliński ^a^*

^a^ Department of Water Protection Engineering and Environmental Microbiology, Faculty of Geoengineering, University of Warmia and Mazury in Olsztyn, Prawocheńskiego 1, 10-720 Olsztyn, Poland

^b^ Department of Immunology of Infectious Diseases, Hirszfeld Institute of Immunology and Experimental Therapy, Polish Academy of Sciences, Weigla 12, 53-114 Wroclaw, Poland

* Correspondence: ewa.korzeniewska@uwm.edu.pl

**Table S1. Sequences of the primers and PCR conditions used in the study.**

| Target gene | Primer sequence (5’-3’) | Amplicon  size  (bp) | Annealing  temperature in standard PCR  (°C) | Reference |
| --- | --- | --- | --- | --- |
| 16S *r*RNA | CGGTGAATACGTTCYCGG  GGWTACCTTGTTACGACTT | 1465 | 56 | Gillan et al.  (1998) |
| *uid*A | ATGGAATTTCGCCGATTTTGC  ATTGTTTGCCTCCCTGCTGC | 166 | 60 | Heijnen and Medema  (2006) |
| *usp*A | CCGATACGCTGCCAATCAGT  ACGCAGACCGTAGGCCAGAT | 884 | 56 | Chen and Griffiths  (1998) |
| *yaiO* | TGATTTCCGTGCGTCTGAATG  ATGCTGCCGTAGCGTGTTTC | 115 | 58 | Molina et al.  (2015) |
| *chu*A | GACGAACCAACGGTCAGGAT  TGCCGCCAGTACCAAAGACA | 279 | 59 | Clemont et al., (2000) |
| TspE4.C2 | GAGTAATGTCGGGGCATTCA  CGCGCCAACAAAGTATTACG | 152 |  |  |
| *yai*A | TGAAGTGTCAGGAGACGCTG  ATGGAGAATGCGTTCCTCAAC | 211 |  |  |
| ERIC | TGTAAGCTCCTGGGGATTCAC  AAGTAAGTGACTGGGGTGAGCG | 50-4200 | 52/65 | Versalovic  (1991) |
| *hly*D | CTCCGGTACGTGAAAAGGAC  GCCCTGATTACTGAAGCCTG | 904 | 50 | Rodriguez-Siek et al. (2005) |
| *eae* | CTGAACGGCGATTACGCGAA  CCAGACGATACGATCCAG | 917 | 58 | Aranda et al.  (2004) |
| *bfp*A | AATGGTGCTTGCGCTTGCTGC  GCCGCTTTATCCAACCTGGTA | 326 |  |  |
| CVD432 | CTGGCGAAAGACTGTATCAT  CAATGTATAGAAATCCGCTGTT | 630 |  |  |
| LT gene | GGCGACAGATTATACCGTGC  CGGTCTCTATATTCCCTGTT | 450 | 50 |  |
| ST gene | ATTTTTMTTTCTGTATTRTCTT  CACCCGGTACARGCAGGATT | 190 |  |  |
| *ipa*H | GTTCCTTGACCGCCTTTCCGATACCGTC  GCCGGTCAGCCACCCTCTGAGAGTAC | 600 |  |  |
| *stx*1 | ATAAATCGCCATTCGTTGACTAC  AGAACGCCCACTGAGATCATC | 180 |  |  |
| *stx*2 | GGCACTGTCTGAAACTGCTCC  TCGCCAGTTATCTGACATTCTG | 255 |  |  |
| *fim*H | TGCAGAACGGATAAGCCGTGG  GCAGTCACCTGCCCTCCGGTA | 506 | 65 | Le Bouguenec et al. (1992) |
| *iro*N | AAGTCAAAGCAGGGGTTGCCCG  GACGCCGACATTAAGACGCAG | 665 | 50 | Johnson et al.  (2000) |
| *sfa* | CTCCGGAGAACTGGGTGCATCTTAC  CGGAGGAGTAATTACAAACCTGGCA | 410 | 63 | Yamamoto et al.  (1995) |
| *pap*C | GACGGCTGTACTGCAGGGTGTGGCG  ATATCCTTTCTGCAGGGATGCAATA | 328 |  |  |
| *bla*_TEM_ | AGTGCTGCCATAACCATGAGTG  CTGACTCCCCGTCGTGTAGATA | 431 | 61 | Kim et al.  (2009) |
| *bla*_SHV_ | GATGAACGCTTTCCCATGATG  CGCTGTTATCGCTCATGGTAA | 214 |  |  |
| *bla*_OXA_ | ATTATCTACAGCAGCGCCAGTG  TGCATCCACGTCTTTGGTG | 296 |  |  |
| *bla*_CTX-M_ | ATTATCTACAGCAGCGCCAGTG  TGCATCCACGTCTTTGGTG | 501 |  |  |
| *tet*(A) | GCTACATCCTGCTTGCCTTC  GCATAGATCGCCGTGAAGAG | 210 | 55 | Ng et al.  (2001) |
| *tet*(B) | TCATTGCCGATACCACCTCAG  CCAACCATCATGCTATTCCATCC | 659 |  |  |
| *tet*(M) | GTGGACAAAGGTACAACGAG  CGGTAAAGTTCGTCACACAC | 406 |  |  |
| *tet(L*) | TCGTTAGCGTGCTGTCATTC  GTATCCCACCAATGTAGCCG | 267 |  |  |
| *tet*(K) | TCGATAGGAACAGCAGTA  CAGCAGATCCTACTCCTT | 169 |  |  |


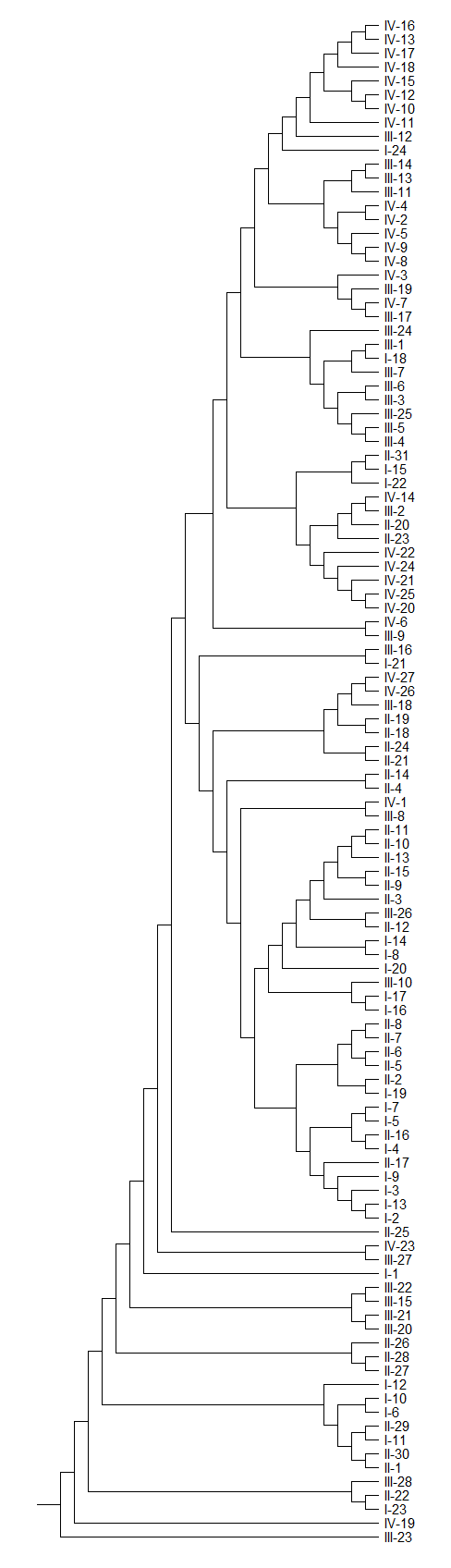

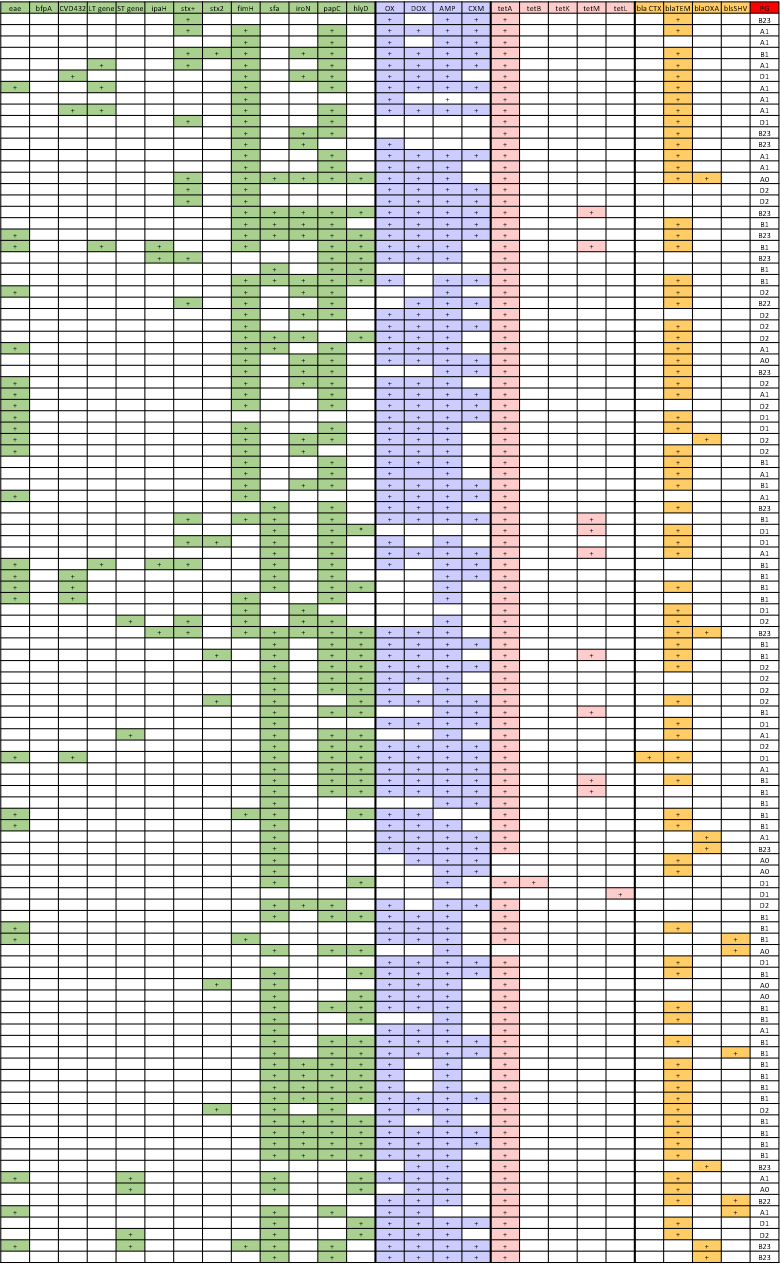


**Fig. S1. Phylogenetic trees of *E.coli* strains isolated from river water**. I-IV – sampling sites – the river which receive effluents from WWTP; 1-28 – number of strains isolated from each sampling site.


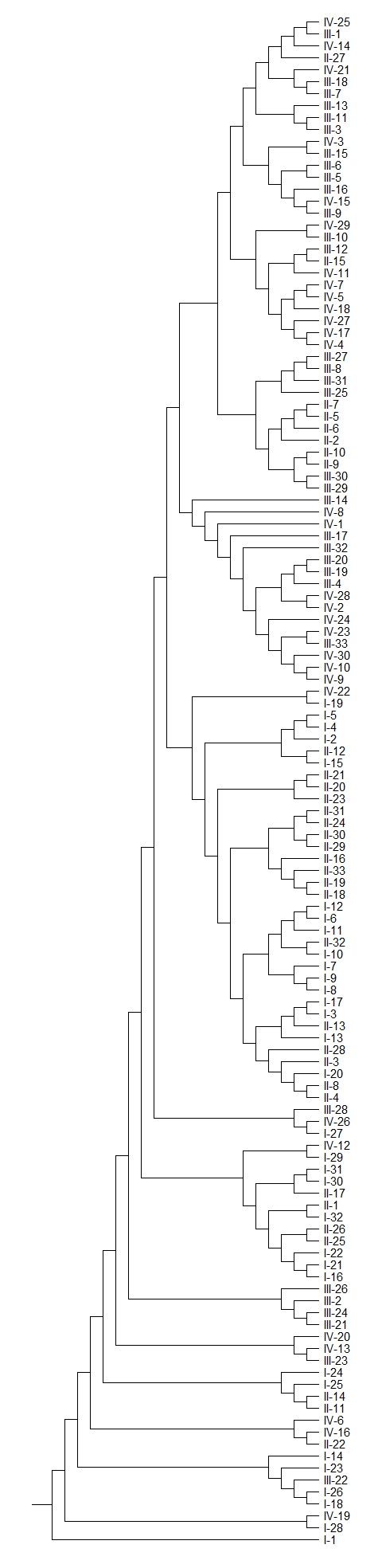

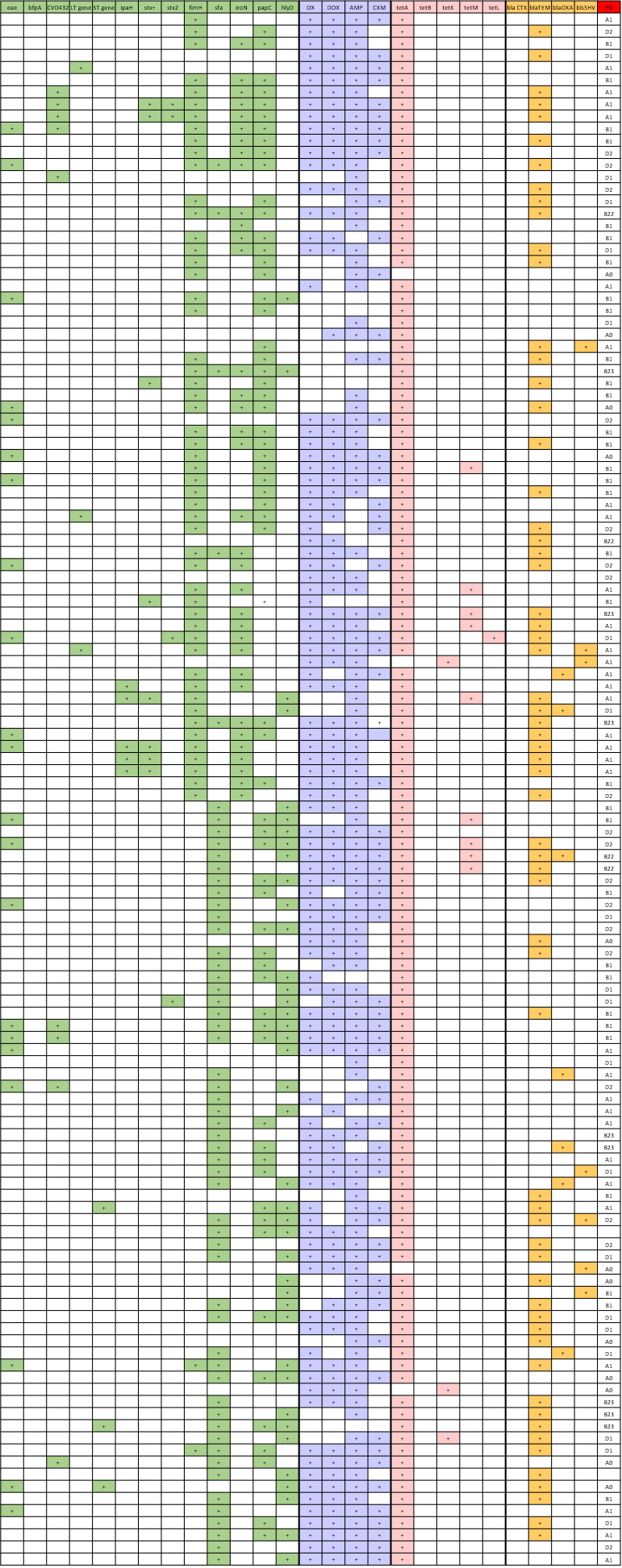


**Fig. S2. Phylogenetic trees of *E.coli* strains isolated from wastewater** I-IV – sampling sites – the WWTPs; 1-28 – number of strains isolated from each sampling site.

**References**

1. Aranda KR, Fagundes-Neto U, Scaletsky IC. Evaluation of Multiplex PCRs for Diagnosis of Infection with Diarrheagenic Escherichia coli and Shigella spp. J. Clin. Microbiol. 2004;42,5849-53.
2. Chen J, Griffiths MW. PCR differentiation of Escherichia coli from other gram-negative bacteria using primers derived from the nucleotide sequences flanking the gene encoding the universal stress protein. Lett. Appl. Microbiol. 1998;27, 369-71
3. Clermont O, Bonacorsi S, Bingen E. Rapid and Simple Determination of the Escherichia coli Phylogenetic Group. Appl. Environ. Microbiol. 2000;66,4555–4558.
4. Gillan DC, Speksnijder AG, Zwart G, De Ridder C. Genetic diversity of the biofilm covering Montacuta ferruginosa (Mollusca, bivalvia) as evaluated by denaturing gradient gel electrophoresis analysis and cloning of PCR-amplified gene fragments coding for 16S rRNA. Appl Environ Microbiol. 1998;64,3464-3472.
5. Heijnen L, Medema G. Quantitative detection of E. coli O157 and other shiga toxin producing E. coli in water samples using a culture method combined with real-time PCR. J. Water Health. 2006;4,487-98.
6. Johnson JR, Russo TA, Tarr PI, Carlino U, Bilge SS, Vary JC. et al. Molecular epidemiological and phylogenetic associations of two novel putative virulence genes, iha and iroN (E. coli), among Escherichia coli isolates from patients with urosepsis. Infect. Immun. 2000;68,3040-7.
7. Kim J, Jeon S, Rhie H, Lee B, Park M, Lee H. et al. Rapid Detection of Extended Spectrum β-Lactamase (ESBL) for Enterobacteriaceae by use of Multiplex PCR-based Method. Infect. Chemother. 2009;41,181-184.
8. Le Bouguenec C, Archambaud M, Labigne A, Rapid and specific detection of the pap, afa, and sfa adhesin-encoding operons in uropathogenic Escherichia coli strains by polymerase chain reaction. J. Clin. Microbiol. 1992;30,1189–1193.
9. Molina F, López-Acedo E, Tabla R, Roa I, Gómez A, Rebollo JE. Improved detection of Escherichia coli and coliform bacteria by multiplex PCR. BMC Biotechnol. 2015;15,48.
10. Ng LK, Martin I, Alfa M, Mulvey M. Multiplex PCR for the detection of tetracycline resistant genes. Mol. Cell Probes. 2001;15,209-15.
11. Rodriguez-Siek KE, Giddings CW, Doetkott C, Johnson TJ, Fakhr MK, Nolan LK, Comparison of Escherichia coli isolates implicated in human urinary tract infection and avian colibacillosis. Microbiology. 2005;151, 2097-2110,
12. Versalovic J, Koeuth T, Lupski JR. Distribution of repetitive DNA sequences in eubacteria and application to fingerprinting of bacterial genomes. Nucleic Acids Res. 1991;19,6823-6831.
13. Yamamoto S, Terai A, Yuri K, Kurazono H, Takeda Y, Yoshida O. Detection of urovirulence factors in Escherichia coli by multiplex polymerase chain reaction. FEMS Immunol. Med. Microbiol. 1995;12,85–90.
